# 3D printing-assisted extended lateral approach for displaced intra-articular calcaneal fractures: a systematic review and meta-analysis

**DOI:** 10.1186/s13018-021-02832-5

**Published:** 2021-11-18

**Authors:** Guang Shi, Wei Liu, Ying Shen, Xiyu Cai

**Affiliations:** Department of Orthopedic, The Fifth Affiliated Hospital of Sun Yat-sen University, Zhuhai, 519000 Guangdong Province China

**Keywords:** Calcaneal, Fracture, 3D printing, Meta-analysis

## Abstract

**Background:**

Three-dimensional (3D) printing technology has developed rapidly in orthopaedic surgery and effectively achieves precise and personalized surgery. The purpose of this meta-analysis was to assess the efficacy of 3D printing technology in the management of displaced intra-articular calcaneal fractures (DICFs) by extended lateral approach (ELA).

**Methods:**

We searched PubMed, Web of Science, Embase, Cochrane Library, CNKI, VIP, and VANFUN databases were searched up to October 2020. All clinical studies comparing traditional surgery and 3D printing-assisted surgery in the management of DICFs were obtained, evaluating the quality of the included studies and extracting data. For each study, we assessed odds ratios (ORs), standard mean difference (SMD), and 95% confidence interval (95% CI) to assess and synthesize the outcomes.

**Results:**

Three RCTs and nine retrospective studies involving 732 patients were included met our inclusion criteria with 366 patients in the 3D group and 366 patients in the conventional group. The meta-analysis showed that there were significant differences of the operative time in the 3D group [SMD =  − 1.86, 95% CI (− 2.23, − 1.40), *P* < 0.001], intraoperative blood loss [SMD =  − 1.26, 95% CI (− 1.82, − 0.69), *P* < 0.001], the number of intraoperative X-ray exposures [SMD =  − 0.66, 95% CI (− 1.20, − 0.12), *P* < 0.001], postoperative complications [OR = 0.49, 95% CI (0.31, 0.79), *P* < 0.001], excellent and good rate of calcaneal fracture outcome [OR = 4.09, 95% CI (2.03, 8.22), *P* < 0.001].

**Conclusion:**

The current study indicates that 3D printing-assisted ELA surgery showed a better rate of excellent and good outcome, shorter operation time, less intraoperative blood loss, fewer intraoperative fluoroscopies, fewer complications. Besides, there is still a need for large-sample, high-quality, long-term randomized controlled trials to confirm the conclusion.

## Introduction

Calcaneus fractures make up about 2% of all fractures [1], most of which are caused by severe high-energy injuries such as falls from height or road traffic accidents [2, 3], and 70 to 75% are intra-articular fractures [4]. The management of calcaneal intra-articular fractures has been controversial [5], and various complex DICFs, after surgery, there is still a risk of deformity and complications such as traumatic arthritis, leg and foot stiffness, deformation, and pain [6, 7]. Traditional surgery usually performs preoperative planning based on X-ray and CT, which cannot involve the 3D structure of the fracture, and only have a limited understanding of the fracture patterns. The increased surgery's invasiveness inevitably leads to unnecessary tissue damage and increases intraoperative bleeding and operation time [8]. In consequence, the outcome of that management is still far from satisfaction.

With the development of digital medicine, more fracture relative information has been available in recent years, and 3D measurements based on CT processing are highly reliable. Preoperative 3D modelling allows for more effective diagnosis and simulates the surgical procedure, 3D printing technology, and digital image processing allow for assessing the post repositioning situation [9, 10]. 3D printing technology has become one of the leading advanced methods of preoperative planning. Several studies reported that 3D printing led to fewer misplacements and errors during the procedure [11, 12]. Some studies point to using 3D technology to reduce the number of postoperative complications and improve the safety of surgical patients [13, 14].

However, an study reported that 3D printing to assist in managing DICFs cannot improve postoperative function compared to routine treatment [15]. Meanwhile, the results in comparing the application of 3D printing-assisted ELA with conventional ELA for DICFs are not entirely consistent in terms of excellent rates of the outcome, operative time, intraoperative bleeding, the number of X-ray exposures, postoperative complications [16, 17]. Furthermore, previous meta-analyses indicated no significant results regarding the rate of excellent and good outcomes, and complications [18]. Considering the above controversies and uncertainties, we conducted a meta-analysis of the effectiveness and safety of applying 3D printing for DICFs to provide a basis for clinical decision-making.

## Materials and methods

This systematic review was designed, undertaken, and reported based on the Preferred Reporting Items for Systematic Reviews and Meta-Analyses (PRISMA) [19] and the Cochrane Collaboration guidelines.

### Search strategy

PubMed, Web of Science, Embase, Cochrane Library, CNKI, VIP, and VANFUN databases were searched up to October 2020. All clinical studies comparing conventional surgery and 3D printing-assisted surgery in the management of DICFs by ELA were obtained. The search terms for the Chinese and English databases were “calcaneal fractures” and “3D printing”. Two researchers independently screened the literature for inclusion and exclusion criteria according to the Cochrane Handbook 5.2 evaluation criteria, read the title and/or abstract information for initial screening, then read the full text to eliminate further literature that cannot meet the criteria and cross-checked the results. The final data was extracted from the literature that met the criteria. In case of disagreement between two researchers, a third researcher is involved in the discussion and negotiates the decision, and if necessary, the authors of the literature may be contacted to clarify further information about the study. Following the PICOS (Participants, Interventions, Comparisons, Outcomes, and Study design) principle, the key search terms included (*P*) patients with calcaneal fracture; (*I*) patients were treated by 3D printing-assisted ELA; (C/O) the primary outcome indicator was the rate of excellent and good outcome, and the secondary outcome indicators were operative time, intraoperative bleeding, number of intraoperative X-rays, and postoperative complications. Inclusion and exclusion criteria applied to articles identified in the literature are shown in Table 1.

**Table 1:** Inclusion and exclusion criteria

*Inclusion criteria*
1. Type of study Clinical study on preoperative planning of DICFs using ELA with the aid of 3D printing technology, in Chinese and English only
2. Study population Inclusion of fractures of the calcaneal diagnosed by X-ray and CT, fracture line involving the articular surface, Sanders classification [35] (type II, III, IV), age ≥ 18 years, no gender restriction
3. Interventions 3D printing-assisted versus non-3D printing-assisted ELA for DICFs
4. Outcome indicators excellent rate of calcaneal fracture outcomes (evaluated using the Maryland scale [36]), operative time, intraoperative bleeding, number of X-ray fluoroscopies, postoperative complications
*Exclusion criteria*
1. Data were not authentic or detailed and could not be extracted
2. Exclusion of those with other incisions or additional other incisions
3. Exclusion of those with other serious injuries (e.g., multiple fractures), lesions (e.g., arthritis), and a history of surgery (e.g., revision in this case), serious systemic diseases (e.g., inflammatory or metabolic diseases) that prevented surgery, etc

### Data extraction

The trial selection process complied with the Preferred Reporting Items for Systematic Reviews and Meta-Analyses statement [20]. For each included study, two types of information were extracted: basic information and primary study outcome. Basic information relevant to this meta-analysis included: author, year, number of cases, age, fracture subtype, study type, and follow-up time. The primary outcome indicator and the secondary outcome indicators: the rate of excellent and good outcome, the duration of operation time, intraoperative blood loss, number of fluoroscopies, complications rate.

### Risk of bias assessment

Both reviewers independently assessed the risk of bias using the Cochrane Collaboration's Risk of bias tool [21]. Appraisal criteria included: sequence generation for randomization, concealment of allocation, blinding, incomplete outcome data, selective outcome reporting, and other potential sources of bias (for example, an extreme imbalance in baseline patient characteristics). Each of these factors was recorded as yes (‘low’ risk of bias), no (‘high-risk), or unclear with a summary provided in table format (see the Characteristics of included studies). Where data were ambiguous, we contacted authors for clarification, where possible. After this process, each paper was graded as low, unclear, or high risk of bias.

### Statistical analysis

Statistical analysis of the data was performed Rev Man 5.4 software provided by the Cochrane Collaboration Network. The results of each study were tested for heterogeneity using *I*^2^ and *P* values. If (*P* ≥ 0.05, *I*^2^ ≤ 50%), there was homogeneity between the results of the studies, and meta-analysis was performed using a fixed-effects model. If the results of each study (*P* < 0.05, *I*^2^ > 50%), there was substantial heterogeneity between the studies, and meta-analysis was performed using a random-effects model. Standard mean differences (SMD) and 95% confidence intervals (CI) were used for measurement data, and ratio (OR) and 95% CI were used for dichotomous variable data. *P* < 0.05 was considered a statistically significant difference.

## Results

### Literature search

**Fig. 1:**
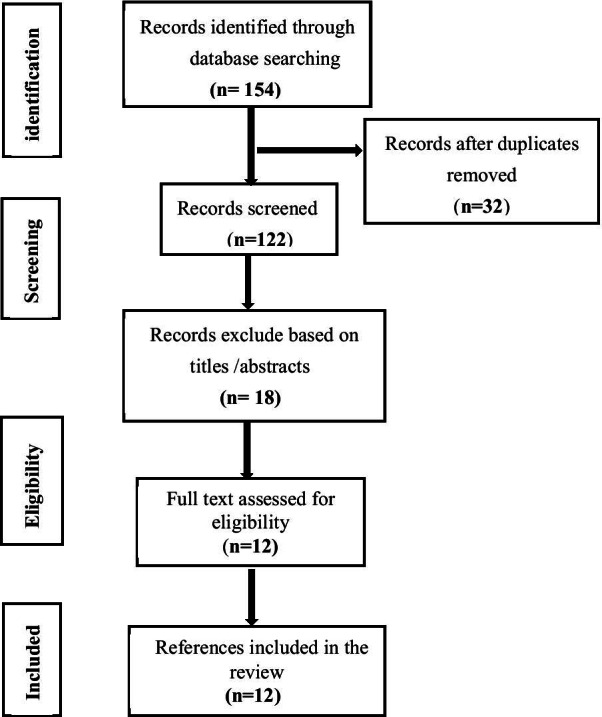
Literature search and selection strategy

The database was searched to obtain 154 relevant papers. After importing them into EndNote X9 software, 122 were checked, 35 were obtained after reading the titles and abstracts, and 12 studies were finally included after reading the full text. The literature selection process is shown in Fig. 1. Seven hundred thirty-two patients were included, 366 patients in 3D printing technology-assisted ELA and 366 patients in conventional ELA.

### Characteristics of the included studies and quality and bias assessment

**Fig. 2:**
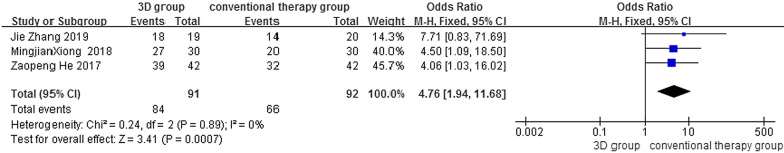
Forest plot for sensitivity analysis the rate of excellent and good outcome

**Fig. 3:**
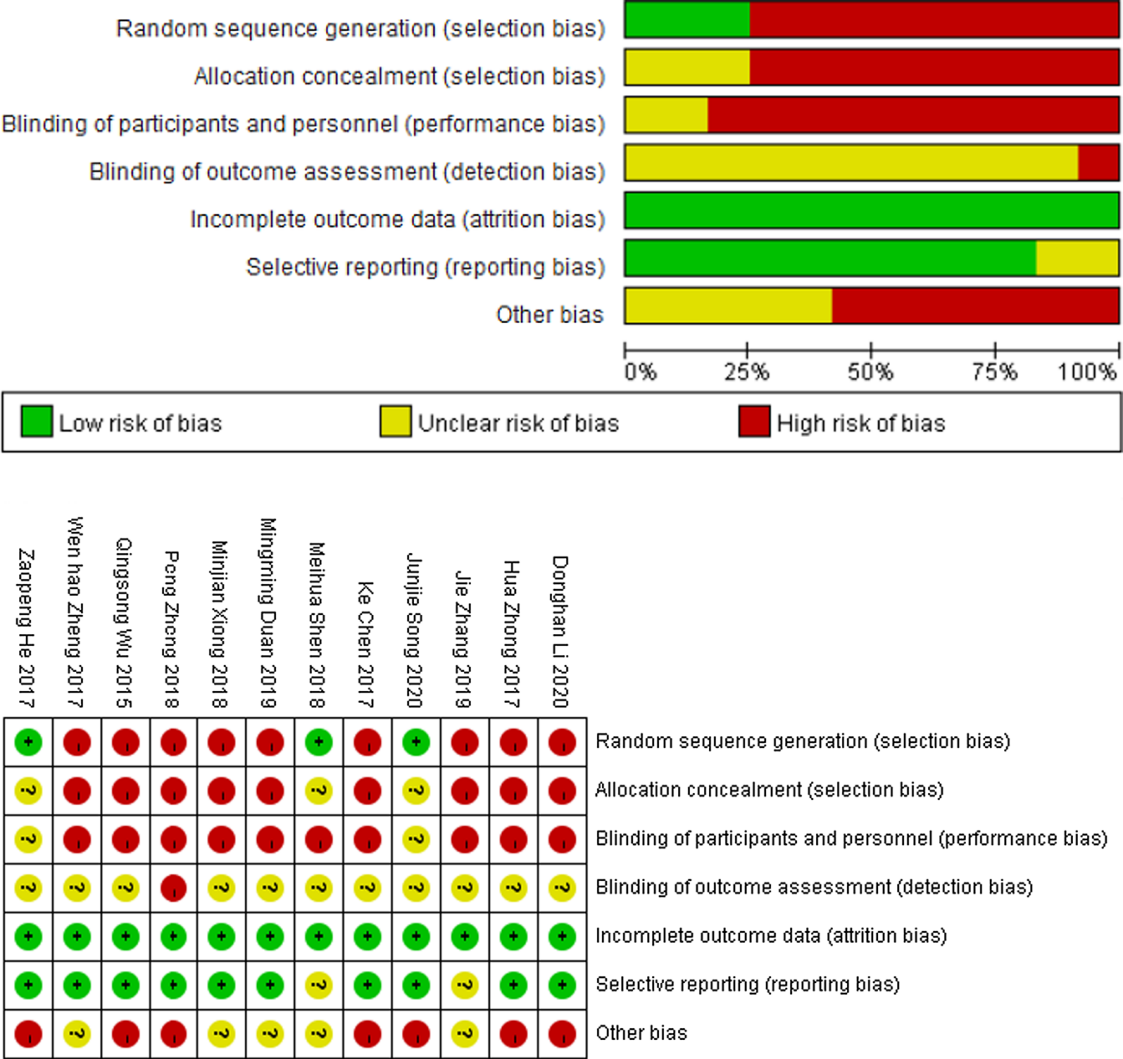
Risk bias of the included research

An assessment of study quality and risk of bias was performed Rev Man 5.4 software offset risk table. Sensitivity analyses were performed in the excellent calcaneal fracture outcome rate. The analysis results showed that the overall results did not show significant changes and the results were relatively stable so that individual studies could be considered not to have a significant effect on the overall results. When the literature with the greatest weight was excluded, the combined effect size (OR = 4.76), 95% CI (1.94, 11.68), and the results did not show a significant change (Fig. 2). Risk of bias graph: review authors’ judgments about each risk of bias item presented as percentages across all included studies (Fig. 3). Table 2 summarizes the basic information for each study.

**Table 2:** The basic characteristics description of included studies

Authors	Years	Patients cases	Average age	Sanders typing	Type of study	Follow-up time	Outcomes
		Man/female	3D/conventional group	II/III/IV			
Chen et al. [37]	2017	53/27	38.00 ± 8.30/36.80 ± 8.20	52/28/0	Retro	20 weeks	a c d
Duan et al. [38]	2019	26/19	35.70 ± 8.40/36.50 ± 8.20	31/14/0	Retro	Not mentioned	a b
He et al. [39]	2017	39/33	38.50 ± 7.60/38.20 ± 6.70	0/25/11	RCT	Not mentioned	a b c e
Li et al. [16]	2020	67/38	36.82 ± 11.02/36.17 ± 10.13	0/58/47	Retro	6 months	a b c d e
Shen et al. [17]	2018	26/10	36.82 ± 11.02/36.17 ± 10.13	0/17/19	RCT	15 months	a b d
Song et al. [40]	2020	32/24	37.02 ± 8.15/36.82 ± 7.76	0/35/21	RCT	6 months	c e
Zheng et al. [41]	2017	44/31	46.70 ± 6.20/44.50 ± 8.00	28/29/18	Retro	15 months	a b c d e
Wu et al. [42]	2015	27/17	36.68 ± 11.26/35.68 ± 11.32	22/22/0	Retro	Not mentioned	a b d e
Xiong et al. [43]	2018	37/23	38.60 ± 7.17/38.56 ± 7.52	0/41/19	Retro	Not mentioned	a b c e
Zhong et al. [44]	2017	23/13	40.0 ± 10.00/39.00 ± 8.00	13/16/7	Retro	10–12 months	a c e
Zhang et al. [10]	2019	23/16	37.50 ± 7.40/36.20 ± 8.50	26/13/0	Retro	6 months	a b c e
Zheng et al. [45]	2018	39/33	37.50 ± 6.50/37.45 ± 6.55	0/43/23	Retro	Not mentioned	a b c e

Outcome indicators: a. operation duration; b. intraoperative blood loss; c. postoperative complications; d. the number of X-ray exposures; e. the rate of excellent and good outcome

### The rate of excellent and good outcome

**Fig. 4:**
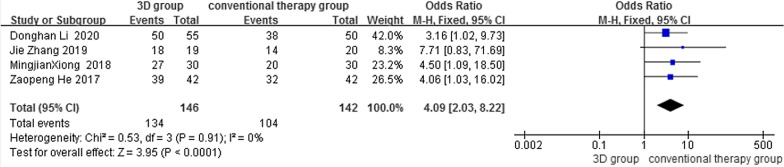
Forest plot for excellent postoperative outcomes in the 3D group versus the conventional group

Four studies reported postoperative outcomes based on Maryland score results of the heterogeneity analysis showed homogeneity between the studies (*P* > 0.05, *I*^2^ = 0%), so a fixed-effects model was used to analyze the results. The results showed a statistically significant difference between the two groups: OR = 4.09, 95% CI (2.03, 8.22), *Z* = 3.95 (Fig. 4).

### Operation duration

**Fig. 5:**
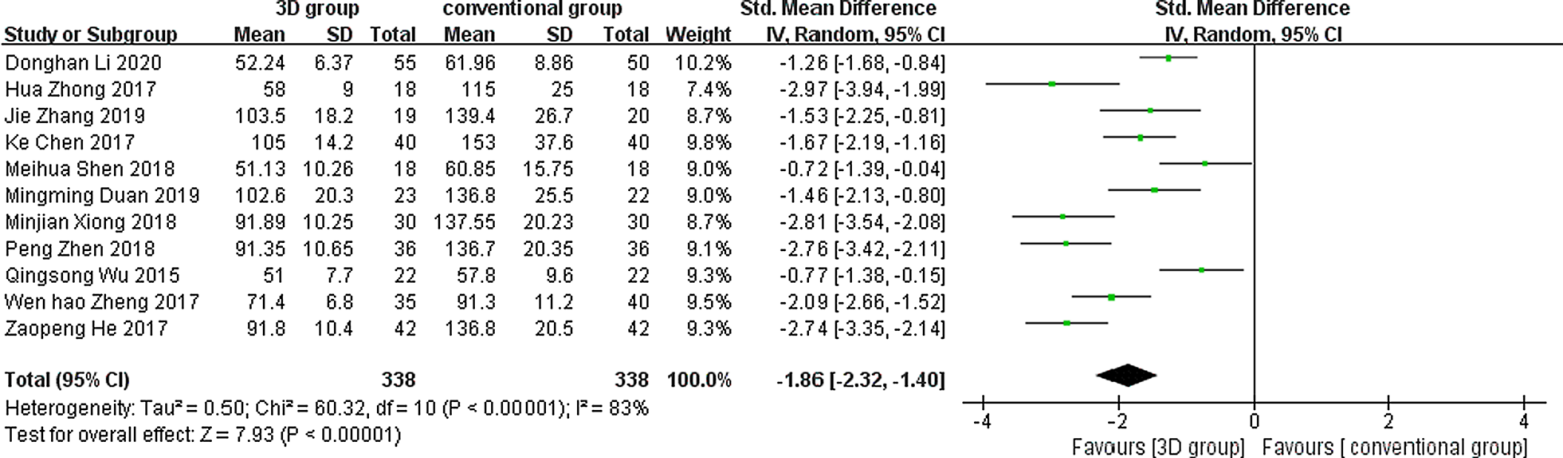
Forest plot of operation duration for the 3D group compared to the conventional group

Eleven studies reported the duration of operation time, with significant heterogeneity between studies (*P* < 0.001, *I*^2^ = 96%), so a random-effects model was used for statistical analysis. The results showed a statistically significant difference between the two groups: SMD =  − 1.86, 95% CI (− 2.23, − 1.40), *Z* = 7.93, *P* < 0.05 (Fig. 5).

### Intraoperative blood loss

**Fig. 6:**
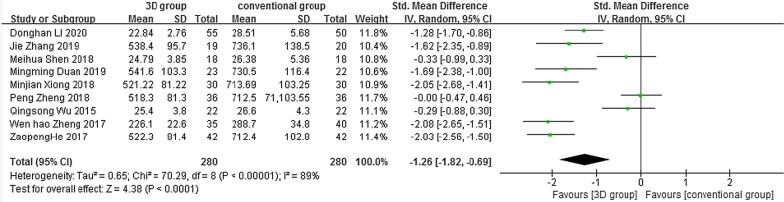
Forest plot of intraoperative bleeding in the 3D group compared to the conventional group

Nine studies reported intraoperative blood loss, and the results of the heterogeneity analysis showed significant heterogeneity between the nine studies (*P* < 0.001, *I*^2^ = 89%), so a random-effects model was chosen for statistical analysis and combined. The research showed a statistically significant difference between the two groups: SMD =  − 1.26, 95% CI (− 1.82, − 0.69), *Z* = 4.38, *P* < 0.05 (Fig. 6).

### Number of intraoperative X-ray exposures

**Fig. 7:**
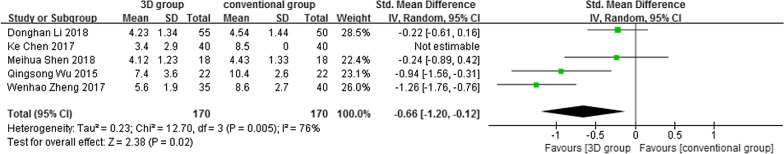
Forest plot of the number of intraoperative X-ray exposures in the 3D group compared to the conventional group

Five studies reported the number of intraoperative X-ray exposures. The heterogeneity analysis results showed heterogeneity between the studies (*P* < 0.001, *I*^2^ = 76%), so a random-effects model was chosen for statistical analysis and combined. The research showed a statistically significant difference in the number of intraoperative X-ray exposures between the two groups: SMD =  − 0.66, 95% CI (− 1.20, − 0.12), *Z* = 2.38, *P* < 0.05 (Fig. 7).

### Postoperative complications

**Fig. 8:**
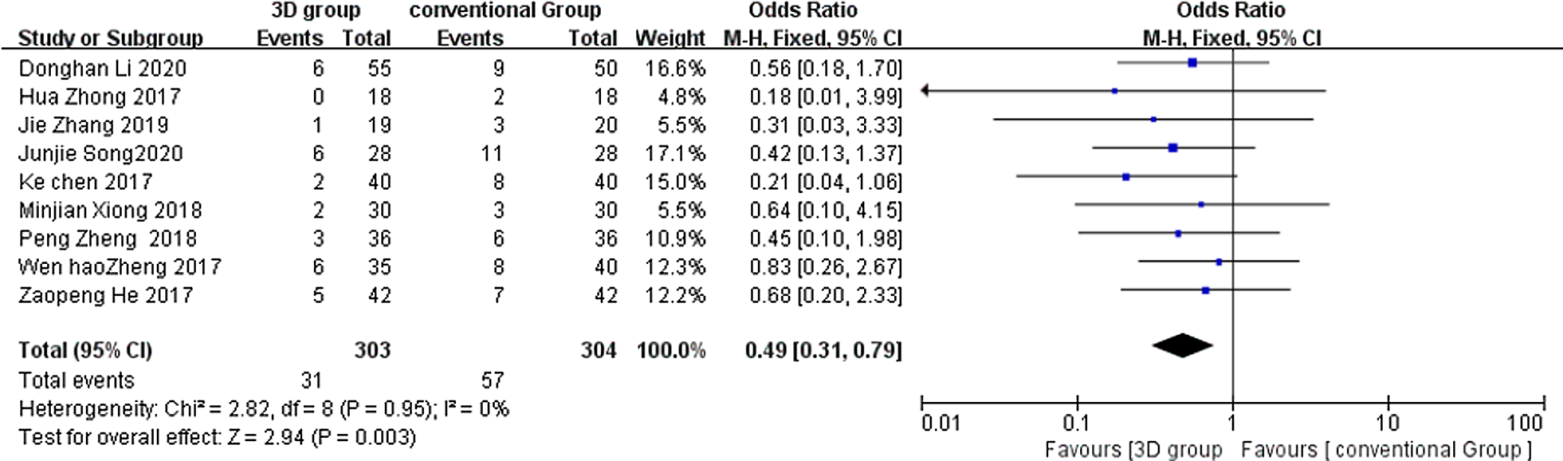
Forest plot of postoperative complications in the 3D group compared to the conventional group

Nine studies reported postoperative complications and the results of the heterogeneity analysis showed homogeneity between the nine studies (*χ*^2^ = 2.82, *P* = 0.95, *I*^2^ = 0%), so a fixed-effects model was chosen for statistical analysis and combined. The results of the analysis showed a statistically significant difference in the incidence of postoperative complications between the two groups: OR = 0.49, 95% CI (0.31, 0.79), *Z* = 2.94, *P* = 0.003 < 0.05 (Fig. 8).

## Discussion

DICF can cause displacement of the articular surface and is a mighty destructive fracture. The subtalar joint, in particular, may be severely disrupted [1]. DICFs remains one of the most challenging problems to manage [22, 23]. Therefore, understanding the morphological characteristics of calcaneal fractures may conduce to treatment. The 3D printing-assisted surgery was critical for surgeons to understand the fracture patterns fully, make a detailed and reliable preoperative plan, and perform individualized therapy for patients. Although there had been some retrospective articles on 3Dprinting-assisted DICFs surgery, there was still a lack of future high-quality articles for further analysis. The current study indicates that 3D printing-assisted ELA surgery outperforms conventional surgery, with shorter operation duration, fewer complications, less intraoperative blood loss, less intraoperative fluoroscopies. Furthermore, our research has shown that the application of 3D printing-assisted ELA can improve the excellent and good rate of calcaneal fracture outcomes.

The excellent and good rate of calcaneal fracture outcome was evaluated according to the Maryland score criteria. Zhang et al. reported that the application of 3D printing-assisted lateral incision cannot improve the excellent and good rate of outcome compared to the conventional group for DICFs, and the difference between the total incidence of the two groups was not statistically significant (*P* > 0.05). This meta-analysis revealed that there was a significant difference in an excellent and good rate of outcome [OR = 4.09, 95% CI (2.03, 8.22)], between the two groups. We found that the application of 3D printing to assist ELA surgical treatment could significantly improve the excellent and good rate of calcaneal fracture outcome.

Postoperative complications included infection, persistent deformity, incongruent joint surfaces with a stiff, painful, deformed foot, osteoarthritis of the subtalar joint are common outcomes [6]. Li et al.[16] reported that no statistically significant difference in postoperative complications and intraoperative X-ray fluoroscopy with 3D printing-assisted ELA compared to the conventional group for DICFs (*P* > 0.05). Zhang et al. also concluded that there was no difference in the efficacy of applying 3D printing to treat DICFs. The meta-analysis revealed that there was a significant difference in postoperative complications [OR = 0.49, 95% CI (0.31, 0.79)], especially wound infection. We believed that the application of 3D printing to assist ELA surgical treatment could significantly reduce the occurrence of postoperative complications. We were considering that being a small sample of studies and mostly non-randomized controlled trials significantly reduces the credit quality rating of the studies. Meta-analysis and systematic evaluation have not been used to compare the efficacy of the 3D printing-assisted ELA to conventional treatment of DICFs, and this is the first time we have used meta-analysis to evaluate the efficacy of the 3D printing-assisted ELA in the treatment of DICFs. The evidence for the results of the meta-analysis is largely conclusive, and further studies are unlikely to reverse the results. Besides, sensitivity analyses confirmed that the advantages of 3D printing-assisted ELA are still stable, which may contribute to the spread of 3D printing in the treatment of DICFs.

There have been many studies confirming the value of 3D printing for applications. 3D printing is emerging as a powerful tool for tissue engineering by enabling 3D cell culture within complex 3D biomimetic architectures [24]. The 3D-printed model offers the benefits of haptic feedback, direct manipulation, and enhanced understanding of cardiovascular anatomy and underlying pathology. 3D printing span from diagnostic assistance and optimization of management algorithms in complex cardiovascular diseases to planning simulating surgical and interventional procedures [25]. The use of 3D printing is proving to be more effective than traditional 2D imaging models in surgical procedures [26]. Chung et al. [27] showed that the application of 3D printing technology to assist in the internal fixation of steel plates can be more beneficial. Misselyn et al. [28] concluded that the 3D printing improves interobserver agreement in assessing calcaneal fractures. 3D printing has become one of the most revolutionary and powerful tools [15]. However, Xu et al. [29] and Chen et al. [30] concluded that 3D printing technology requires a high level of professional expertise and increases the cost of treatment for patients, limiting its widespread availability. The cost of this technique is another reason limiting its diffusion [31–33]. Indeed, the cost is often a concern when new and expensive technologies are introduced into medical practice. However, this may be resolved in the coming years as the cost of 3D printing decreases. Indeed, the cost is often a concern when new and expensive technologies are introduced into medical practice. A systematic evaluation noting the additional cost and time required to manufacture devices with current 3D technology is still widely used in hospitals. Still, guidelines need to be developed to improve the reporting of experience with 3D printing in orthopedic surgery is highly desirable [34]. However, this may be resolved in the coming years as the cost of 3D printing decreases.

Although this study was conducted by comprehensively screening and obtaining all relevant literature, the inclusion–exclusion criteria and the literature quality evaluation system were strictly adhered to ensure the quality of the included literature as much as possible. The results of this Meta-analysis have certain clinical guidance implications. However, this study also has certain limitations: (1) The relatively low quality of the included studies severely reduced the quality level of the evidence and compromised the credibility of the overall pooled effect estimates (2) The inclusion language was restricted to Chinese and English, and literature published in other languages was not included in the study. (3) Preoperative communication satisfaction, incision length, length of stay, fracture healing time, and AOFAS score outcome indicators were not included in the study. (4) Despite this, the sensitivity analysis results were generally consistent with the overall pooled effect, which supports the robustness and reliability of the benefits of 3D printing ELA treatment of DICFs. (5) It is hard to ignore the potential clinical and methodological heterogeneity across included studies due to our systematic review's broad inclusion criteria, although the statistical heterogeneity was low. Conservative estimation of the effect of 3D printing-assisted ELA on the efficacy of calcaneal fracture treatment using a random-effects model.

## Conclusion

The current study indicates that 3D printing-assisted ELA surgery showed a better rate of excellent and good outcome, shorter operation time, less intraoperative blood loss, fewer intraoperative fluoroscopies, fewer complications. Besides, there is still a need for large-sample, high-quality, long-term randomized controlled trials to confirm the conclusion.

## Data Availability

All data generated or used during the study appear in the submitted article.

## Abbreviations

3D: Three-dimensional
ELA: Extended lateral approach
DICFs: Displaced intra-articular calcaneal fractures
